# Behavioural and clinical predictors for Loiasis

**DOI:** 10.7189/jogh.08.010413

**Published:** 2018-06

**Authors:** Johannes Mischlinger, Luzia Veletzky, Gildas B Tazemda-Kuitsouc, Paul Pitzinger, Pierre B Matsegui, Markus Gmeiner, Heimo Lagler, Tamirat Gebru, Jana Held, Benjamin Mordmüller, Michael Ramharter

**Affiliations:** 1 Department of Medicine I, Division of Infectious Diseases and Tropical Medicine, Medical University of Vienna, Vienna, Austria; 2 Centre de Recherches Médicales de Lambaréné, Lambaréné, Gabon; 3 Institut für Tropenmedizin, Universität Tübingen, and German Center for Infection Research, partner site Tübingen, Tübingen, Germany; 4 Bernhard Nocht Hospital for Tropical Diseases, Bernhard Nocht Institute for Tropical Medicine and University Medical Center Hamburg-Eppendorf, Hamburg, Germany; 5 Centre de Recherches Médicales de la Ngounié, Fougamou, Gabon

## Abstract

**Background:**

Loiasis is a vector-borne disease in Central and West Africa. While there is still uncertainty to what extent loiasis is responsible for population morbidity, individuals having both loiasis and onchocerciasis have a high risk of fatal encephalopathy when treatment (ie, ivermectin) for onchocerciasis is given. Therefore it is current policy that communities of high loiasis-burden are excluded from mass drug administration programmes of ivermectin. To address this treatment gap we present diagnostic scores, based on clinical and behavioural predictors that may help to rapidly identify sub-groups with loiasis within high-burden communities.

**Methods:**

A cross-sectional survey was performed in the province of la Ngounie, Gabon between December 2015 and Februrary 2016 and 947 participants of all ages were recruited. Clinical parameters and behavioural exposure factors were ascertained by questionnaire-based interviews. Parasitological analysis of blood samples was performed for *L. loa* detection. Diagnostic scores consisting of clinical and behavioural factors were modelled to predict loiasis in sub-groups residing in endemic regions.

**Results:**

Increasing sylvan exposure was identified as important risk factor for loiasis with adjusted odds ratios of 5.1 (95% confidence interval CI 2.6-9.9) for occasional forest exposure, 11.1 (95% CI 5.4-22.6) for frequent forest exposure and 25.7 (95% CI 12.5-52.9) for intensive forest exposure. Individuals with loiasis were 7.7 (95% CI 5.4-11.0) times more likely to report recurrent pruritus than those without loiasis. Reporting of regular daily exposure to the deep rain forest and recurrent pruritus was 9-fold (positive likelihood ratio 9.18; 95% CI: 6.39-13.18) more prevalent in individuals with loiasis than in controls. Concordantly, the absence of regular weekly forest exposure was associated with extremely low disease-likelihood (negative likelihood ratio 0.09; 95% CI 0.05-0.16).

**Conclusions:**

These composite scores may serve as a simple tool to rapidly identify both those most and those least at risk of disease and may simplify loiasis control activities as well as screening procedures for studies on loiasis. Further, they may aid policy-makers to tailor the delivery of ivermectin mass drug administration for onchocerciasis control programmes more effectively and safely in regions of high loiasis-burden.

Loiasis is a filarial disease which is endemic in Central- and West Africa [1,2]. It is transmitted via the Chrysops fly, which relies on damp woody vegetation as habitat. Pathognomonic symptoms are Calabar swelling, a non-pitting edema occurring mostly over boney prominences, and notion of a worm migrating through the eye [3-6]. Calabar swelling was shown to occur more frequently in Caucasians and history of eye worm more often in Africans [4,7]. In addition, loiasis causes a variety of non-specific symptoms, the most commonly reported one being recurrent pruritus [4,8,9].

*L. loa* has attracted considerable attention in the context of onchocerciasis control programmes [2]. Onchocerciasis is controlled by repeated mass drug administration of ivermectin. Ivermectin may however lead to an unacceptable risk for encephalopathy, when administered to patients with high microfilarial counts of *L. loa* [10]. The identification of communities with high prevalence of *L. loa* microfilariaemia was therefore vital to exclude those communities from mass drug administration programmes. Over the past decade this epidemiological identification of populations has been performed by the “Rapid assessment procedure for *Loa loa”* (RAPLOA) – an assessment relying on the history of eye worm migration [11,12]. However, RAPLOA has not been designed and evaluated for the identification of loiasis in individual patients.

The diagnosis of loiasis in individual patients is based on the detection of the adult worm, microfilariae, or serological tests [2]. Microfilariae can be detected by light microscopy of peripheral blood with peak microfilaraemia occurring around noon. However, a significant proportion of patients may suffer from occult loiasis with an absence of microfilaremia and cross detection of antibodies limits the usefulness of serology [13,14]. Loiasis is therefore difficult to diagnose in endemic settings. The identification of sub-groups most at risk for the disease is therefore of high importance to adequately allocate limited resources for further diagnostic assessments in the health care sector.

To address these diagnostic challenges and to identify high-risk sub-groups, this study aimed to determine predictors for loiasis. Based on the systematic evaluation of predictors diagnostic composite scores were created [15,16].

## METHODS

### Participants

This cross-sectional survey was conducted in rural central Gabon in the province of la Ngounié, a region covered by tropical rain forest [17]. Between December 2015 and February 2016 study teams visited rural villages surrounding the administrative capital Fougamou and actively recruited villagers in this survey. Community leaders were informed one week prior to the screening activities and the entire community was invited to participate.

### Questionnaire for behavioural and occupational predictors

Questionnaire-based interviews were performed to obtain occupational status and the degree of forest exposure. Forest exposure was defined as “none” (no significant activities in the forest), “occasional” (twice per week exposure to forest), “frequent” (daily activities in the forest) and “intensive” (daily exposure to deep tropical rain forest). Either “occasional”, “frequent” or “intensive” exposure was defined as “regular weekly” exposure. These definitions assumed temporal regularity of exposures and were recorded as indicated by the participant.

### Parasitological and clinical definitions

Blood was sampled between 10:00 and 15:00 and thick blood smears of 10 µL were performed and stained with 4% Giemsa solution for 60 minutes for parasitological analysis with light microscopy. Loiasis was defined as either the presence of *L. loa* microfilariae in a blood sample or a positive history of eyeworm as defined by RAPLOA assessment - a case definition of history of eyeworm endorsed by the World Health Organisation [11,12]. Calabar swelling was not part of the case definition, because it was shown previously that its inclusion can lead to a high false positivity rate [11]. When asked for “eyeworm”, concepts of the local languages were used (“DOBBA” in Shira language). Pruritus was chosen as non-specific symptom and was assessed for all participants [4,8,9]. Pruritus was defined as any recurrent itch reported by a patient or caregiver of a child, for which there was no apparent alternative aetiology in medical history and clinical examination. To further assess the distribution of Calabar swelling among different behavioural exposures, history of Calabar swelling was assessed in a sub-sample of the study population. It was defined as the presence of a uni-lateral, recurring, non-pitting edema that stays for multiple days.

### Ethics statement

The study was conducted according to the ethical principles stated in the Declaration of Helsinki, the applicable guidelines for ICH-GCP, and the applicable laws and regulations of Gabon. Individual written informed consent was obtained from all adult participants. For minors, a parent or legal guardian was asked to sign informed consent on their behalf and from minors above the age of 12 additionally assent was obtained. The study was part of two epidemiological studies on malaria that were approved by the independent ethics committee of the Centre de Recherches Médicales de Lambaréné [18].

### Statistical considerations

STATA 13 (StataCorp, College Station, Texas, TX, USA) was used for statistical analyses. Stata-software packages were downloaded to allow the creation of proportional Venn diagrammes and the computation of diagnostic test performance characteristics [19,20]. Performance characteristics were determined for composite exposures on the basis of the presence/absence of pruritus and various degrees of forest exposure. χ^2^-tests were used for proportions, likelihood ratio tests to obtain p-values for logistic regression models and the test of homgeneity was used to discriminate between confounders and effect modifiers. Cochran-Armitage trend test was used for trend analysis for proportions across an ordinal variable [21]. Post-test probabilities were calculated as described by Kent et al. [22]. The STATA ‘direct standardization’ command for survey data was used to estimate the proportion of Calabar swelling in the overall study population. Information on distribution of Calabar swelling among the age-strata in the sub-population was extrapolated to the age-structure of the overall study population. A significance level of two-sided α<0.05 was considered as statistically significant.

## RESULTS

In total 947 participants were recruited with a median age of 22 years (interquartile range (IQR) 8-51) and a male/female ratio of 0.84 (**Table 1**). 289 out of 947 (30.5%) had loiasis according to our case definition and positivity for RAPLOA was the most frequent loiasis-defining characteristic (65.1%; 188/289) (**Table 2**). Prevalence of pruritus was 32.3% (306/947) in the overall study population and 67.2% (636/947) reported being regularly engaged in activities that involved exposure to the forest at least twice weekly. The sub-study assessing Calabar swelling recruited 213 participants (median age 16; IQR 7 - 40, male/female ratio of 0.90). 60/213 (28.2%) had loiasis.

**Table 1:** Baseline characteristics

	Total cohort (N = 947)	Loiasis (n = 289)	Population of Calabar swelling sub-study (n = 213)
**Characteristics**	No. (column %)	No. (row %)	No. (column %)
**Age (years):**			
Median (IQR)	22 (8-51)	51 (32-65)	16 (7-40)
Below 6	160 (16.9%)	2 (1.3%)	44 (20.6%)
6 to 17	272 (28.7%)	26 (9.6%)	65 (30.5%)
18 to 34	147 (15.5%)	52 (35.4%)	37 (17.4%)
35 to 49	120 (12.7%)	60 (50%)	27 (12.7%)
50 to 64	113 (11.9%)	65 (57.5%)	13 (6.1%)
65 or older	135 (14.3%)	84 (62.2%)	27 (12.7%)
**Sex:**			
Male	434 (45.8%)	124 (28.6%)	101 (47.4%)
**Exposure to forest:**			
None	311 (32.8%)	12 (3.9%)	67 (31.5%)
Occasional	237 (25%)	42 (17.7%)	76 (35.7%)
Frequent	173 (18.3%)	82 (47.4%)	42 (19.7%)
Intensive	226 (23.9%)	153 (67.7%)	28 (13.1%)
**RAPLOA:**			
Positive	245 (25.9%)	245 (100%)	46 (21.6%)
**Microfilaraemia:**			
Positive	101 (10.7%)	101 (100%)	24 (11.3%)
**RAPLOA or microfilaraemia:**			
Positive	289 (30.5%)	289 (100%)	60 (28.2%)
**Pruritus:**			
Positive	306 (32.3%)	206 (67.3%)	42 (19.7%)
**History of Calabar swelling:**			
Positive	13.3% (7.1-23.5)*	10 (62.5%)†	16 (7.5%)

RAPLOA – rapid assessment procedure for Loa loa

*Percentage (95% CI) of history of Calabar swelling was estimated through direct standardization of results from the sub-study population (n = 213) using the age-structure of the whole population (N = 947).

†Calculated only for people with loiasis (n = 16) of sub-study population (n = 213).

**Table 2:** Cross-tabulation of loiasis-defining characteristics*

Total N = 947	RAPLOA + (n, row %)	RAPLOA – (n, row %)
Microfilaraemia +	**57 (56.4%)**	**44 (43.6%)**
Microfilaraemia –	**188 (22.2%)**	**658 (77.8%)**


RAPLOA – rapid assessment procedure for Loa loa

*χ^2^ test (*P* < 0.001).

The prevalence of loiasis rises as the exposure to forest becomes more intensive (*P* < 0.001) (**Table 3**). Crude odds ratios (ORs) demonstrate that the odds for loiasis rises by the factor of 5.4 (95% CI 2.7-10.7) for occasional exposure, 22.5 (95% CI 10.4-48.3) for frequent exposure and 52.2 (95% CI 21.7-125.7) for intensive exposure when compared to no forest exposure. Adjusting for sex and age reduces the overall association, however remaining highly statistically significant (*P* < 0.001). Pruritus was more common in persons with loiasis (206/306; 67.3%; *P* < 0.001) with a crude odds ratio of 13.8 (95% CI 9.3-20.6). Adjustment for age and sex reduces the odds ratio but it remains highly statistically significant (OR 7.7, 95% CI 5.4-11.0; *P* < 0.001).

**Table 3:** Predictors for loiasis

Predictors for loiasis	Loiasis (N = 289)	Crude OR (95% CI)	Adjusted OR (95% CI)
**Age (years):**			
Below 6	2 (1.3%)*	1	1
6 to 17	26 (9.6%)*	8.3 (1.9-36.4)	7.9 (1.8-34.3)†
18 to 34	52 (35.4%)*	43.2 (8.7-215.8)	36.1 (7.2-180.6)†
35 to 49	60 (50%)*	79 (13.4-464.8)	57.9 (8.6 – 391.7)†
50 to 64	65 (57.5%)*	107 (16.2-708.6)	69.8 (7.4 – 662.3)†
65 or older	84 (62.2%)*	130.1 (18.8-901.6)	73.9 (5.0 – 1099.9)†
**Sex:**			
Female	165 (32.2%)‡	1	1
Male	124 (28.6%)‡	0.84 (0.6-1.1)	1 (0.7-1.4)§
**Exposure to forest:**			
None	12 (3.9%)*	1	1
Occasional	42 (17.7%)*	5.4 (2.7-10.7)	5.1 (2.6-9.9)||
Frequent	82 (47.4%)*	22.5 (10.4-48.3)	11.1 (5.4-22.6)||
Intensive	153 (67.7%)*	52.2 (21.7-125.7)	25.7 (12.5-52.9)||
**Pruritus:**			
No	83 (12.9%)*	1	1
Yes	206 (67.3%)*	13.8 (9.3-20.6)	7.7 (5.4-11.0)||

OR – odds ratio, CI – confidence interval

*χ^2^ test (*P* < 0.001); Cochran-Armitage test for trend (*P* < 0.001); only ordinal exposures were tested for trend.

†Likelhood ratio test (*P* < 0.001); adjusted for the factor age divided by 10.

‡χ^2^ test (*P* = 0.23).

§ Likelihood ratio test (*P* = 0.96); adjusted for age.

||Likelihood ratio test (*P* < 0.001); adjusted for age and sex.

In the sub-study population 16 participants (7.5%) reported a recent episode of Calabar swelling (**Table 4**). Among participants with loiasis 10 individuals (10/60, 16.7%) reported a history of Calabar swelling (*P* = 0.002). Among all 16 participants with history of Calabar swelling 12 (75%) reported 'regular weekly forest exposure', 2/12 (16.6%) reported occasional, 3/12 (35.0%) frequent and 7/12 (58.3%) intensive exposure. Twelve out of 16 (75.0%) reported pruritus. History of Calabar swelling was not common in pediatric participants but occurred in a frequency of 11.1% to 16.2% in various age groups of adult participants (*P* = 0.012). History of Calabar swelling was more common in male individuals (9.9% vs 5.4% in women), but the association was not significant (*P* = 0.21). The Cochran-Armitage test for trend was highly significant between history of Calabar swelling and the degree of sylvan exposures (*P* = 0.006) and age groups (*P* = 0.001).

**Table 4:** Frequency of history of Calabar swelling in various strata

Characteristics	History of Calabar swelling (N = 16)*
**Age (years):**	
Below 6	0†
6 to 17	1 (1.5%)†
18 to 34	6 (16.2%)†
35 to 49	4 (14.8%)†
50 to 64	2 (15.4%)†
65 or older	3 (11.1%)†
**Sex:**	
Female	6 (5.4%)‡
Male	10 (9.9%)‡
**Exposure to forest:**	
None	4 (6%)§
Occasional	2 (2.6%)§
Frequent	3 (7.1%)§
Intensive	7 (25%)§
**Pruritus:**	
No	4 (2.3%)||
Yes	12 (28.6%)||

*Total of 213 participants included in the Calabar swelling sub-study.

†χ^2^ test (*P* = 0.012); Cochran-Armitage test for trend (*P* = 0.001).

‡χ^2^ test (*P* = 0.21).

§χ^2^ test (*P* = 0.002); Cochran-Armitage test for trend (*P* = 0.006).

||χ^2^ test (*P* < 0.001).

70.9% of people with occasional forest exposure and over 90% with daily exposure reported subsistence activities as reason for forest exposure (**Table 5**). Sylvan subsistence activities increased with age ranging from 10.6% (17/160) in participants below 6 years of age to 88.2% (119/135) in people aged 65 or older. Cochran-Armitage tests for trend were highly significant (*P* < 0.001). There were no differences between the two sexes (*P* = 0.99).

**Table 5:** Factors associated with subsistence activities in the forest

Characteristics	Subsistence activities in the forest (N = 947)
**Age (years):**	
Below 6	17 (10.6%)*
6 to 17	108 (39.7%)*
18 to 34	96 (65.3%)*
35 to 49	103 (85.8%)*
50 to 64	97 (85.8%)*
65 or older	119 (88.2%)*
**Sex:**	
Female	292 (56.9%)†
Male	248 (57.1%)†
**Exposure to forest:**	
None	0*
Occasional	168 (70.9%)*
Frequent	163 (94.2%)*
Intensive	209 (92.5%)*
**Loiasis:**	
Negative	293 (44.5%)*
Positive	247 (85.5%)*

*χ^2^ test (*P* < 0.001), Cochran-Armitage test for trend (*P* < 0.001); only ordinal exposures were tested for trend.

†χ^2^ test (*P* = 0.99).

**Figure 1:**
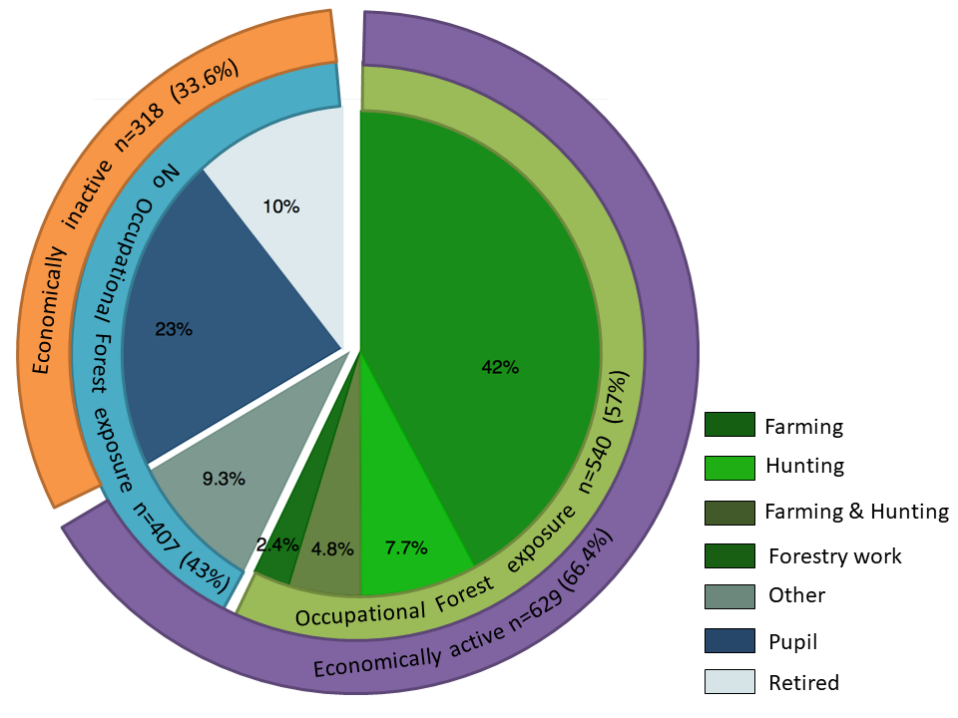
Pie chart, demonstrating occupational status of participants.

Various occupational activities of participants were investigated (**Figure 1**). Among all 947 participants 400 (42.2%) engaged in farming activity, 73 (7.7%) in hunting, 45 (4.8%) in farming and hunting, 23 (2.4%) in forestry work and 88 (9.3%) had other professions that did not involve exposure to the forest. 219/947 (23.1%) pupils and 99/947 (10.5%) retired people reported not to be engaged in any economic or occupational activity.

**Figure 2:**
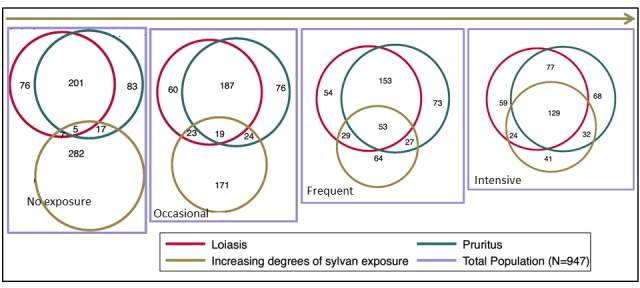
Series of proportional Venn diagrammes showing the overlap of loiasis, pruritus and various degrees of sylvan exposures. From left to right: “No exposure” – no significant activities in the forest; “occasional” – twice per week; “frequent” – daily activities in the forest; “intensive” – daily activities in the deep tropical rain forest.

A series of proportional Venn diagrammes show the overlap between loiasis, pruritus and various degrees of sylvan exposures (**Figure 2**).

“Regular weekly forest exposure” had the highest sensitvity with 95.8% and the most favourable negative likelihood ratio which was 0.09 (95% CI 0.05-0.16). The combined presence of “pruritus” and “intensive forest exposure” had the highest positive likelihood ratio, which was 9.18 (95% CI 6.39-13.18) and an overall area under the receiver operating characteristic (ROC) curve of 0.8 (95% CI 0.76-0.83) (**Table 6** and **Table 7**)

**Table 6:** Performance characteristics of different composite exposures computed from presence of pruritus and sylvan exposure (part 1)

**Composite exposure**	**Sensitivity (%, 95% CI)**	**Specificity (%, 95% CI)**	**PPV (%, 95% CI)**	**NPV (%, 95% CI)**
Regular weekly forest exposure†	95.8% (92.9-97.8)	45.4% (41.6-49.3)	43.6% (39.7-47.5)	96.1% (93.4-98.0)
Occasional forest exposure	14.5% (10.7-19.1)	70% (66.7-73.8)	17.7% (13.1-23.2)	65.2% (61.6-68.7)
Frequent forest exposure	28.4% (23.2-33.9)	86.2% (83.3-88.7)	47.4% (39.8-55.1)	73.3% (70.0-76.3)
Intensive forest exposure	52.9% (47.0-58.8)	88.9% (86.3-91.2)	67.7% (61.2-73.7)	81.1% (78.1-83.9)
(Frequent or intensive) forest exposure	81.3% (76.3-85.6)	75.1% (71.6-78.3)	58.9% (53.9-63.8)	90.1% (87.3-92.5)
Pruritus	71.3% (65.7-76.4)	84.8% (81.8-87.5)	67.3% (61.8-72.5)	87.1% (84.2-89.6)
Pruritus + regular weekly forest exposure	69.6% (63.9-74.8)	87.4% (84.6-89.8)	70.8% (65.1-76.0)	86.7% (83.9-89.2)*
Pruritus + occasional forest exposure	6.6% (4.0-10.1)	96.4% (94.6-97.6)	44.2% (29.1-60.1)	70.1% (67.0-73.1)
Pruritus + frequent forest exposure	18.3% (14.0-23.3)	95.9% (94.1-97.3)	66.3% (54.8-76.4)	72.8% (69.7-75.7)
Pruritus + intensive forest exposure	44.6% (38.8-50.6)	95.1% (93.2-96.7)	80.1% (73.1-86.0)	79.6% (76.7-82.4)
Pruritus + (frequent or intensive) forest exposure	63% (57.1-68.6)	91% (88.6-93.1)	75.5% (69.6-80.8)	84.8% (82.0-87.4)

CI – confidence interval, PPV – positive predictive value, NPV – negative predictive value

*NPV of 97.6% (95.1 - 99.0) if both pruritus & regular weekly forest exposure are reported negative.

†“Regular weekly forest exposure” defined as at least twice per week.

**Table 7:** Performance characteristics of different composite exposures computed from presence of pruritus and sylvan exposure (part 2)*

**Composite exposure**	**LR (+) (ratio, 95% CI)**	**LR (-) (ratio, 95% CI)**	**Area under ROC curve (95% CI)**	**Post-test p (+) (%, 95% CI)**	**Post-test p (-) (%, 95% CI)**
Regular weekly forest exposure	1.76 (1.63-1.89)	0.09 (0.05-0.16)	0.69 (0.68-0.72)	43.6% (39.7-47.5)	3.9% (2.0-6.6)
Occasional forest exposure	0.49 (0.36-0.66)	1.21 (1.13-1.30)	0.41 (0.38-0.44)	17.7% (13.1-23.2)	34.8% (31.3-38.4)
Frequent forest exposure	2.05 (1.57-2.67)	0.83 (0.77-0.90)	0.60 (0.56-0.64)	47.4% (39.8-55.1)	26.7% (23.7-30.0)
Intensive forest exposure	4.77 (3.75-6.08)	0.53 (0.47-0.60)	0.74 (0.71-0.78)	67.7% (61.2-73.7)	18.9% (16.1-21.9)
(Frequent or intensive) forest exposure	3.26 (2.83-3.77)	0.25 (0.19-0.32)	0.75 (0.72-0.77)	58.9% (53.9-63.8)	9.9% (7.5-12.7)
Pruritus	4.69 (3.86-5.70)	0.34 (0.28-0.41)	0.77 (0.74-0.81)	67.3% (61.8-72.5)	12.9% (10.4-15.8)
Pruritus + regular weekly forest exposure	5.51 (4.45-6.84)	0.35 (0.29-0.42)†	0.79 (0.76-0.82)	70.8% (65.1-76.0)	13.3% (10.8-16.1)†
Pruritus + occasional forest exposure	1.80 (1.00-3.24)	0.97 (0.94-1.00)	0.57 (0.50-0.65)	44.2% (29.1-60.1)	29.9% (26.9-33.0)
Pruritus + frequent forest exposure	4.47 (2.87-6.96)	0.85 (0.80-0.90)	0.70 (0.64-0.75)	66.3% (54.8-76.4)	27.2% (24.3-30.3)
Pruritus + intensive forest exposure	9.18 (6.39-13.18)	0.58 (0.52-0.65)	0.80 (0.76-0.83)	80.1% (73.1-86.0)	20.4% (17.6-23.3)
Pruritus + (frequent or intensive) forest exposure	7.02 (5.42-9.10)	0.41 (0.35-0.47)	0.80 (0.77-0.83)	75.5% (69.6-80.8)	15.2% (12.6-18.0)

LR (+) – likelihood ratio for positive test result; LR (-) – likelihood ratio for negative test result; Post-test p (+) – post-test probability for positive result; Post-test p (-) – post-test probability for negative result, ROC – receiver operating characteristic curve, CI – confidence interval

*N.B.: Pre-test probability for loiasis in the study population = 30.5%.

†LR (-) of 0.06 (0.03-0.12) and post-test p (-) of 2.4% (1-0-4.9) if both pruritus & regular weekly forest exposure are reported negative.

## DISCUSSION

Loiasis is a neglected tropical disease that occurs in forested regions of Central and West Africa. This study shows that varying degrees of sylvan exposures are important risk factors for loiasis, a finding supported by the transmission cycle with the *Chrysops* fly serving as the vector and having its habitat in the high-canopied parts of the tropical rain forest [1,2].

The intensity of forest exposure of rural populations in this region is associated with age, sex and socio-economic status. Women engage more often in subsistence agriculture and men more frequently in hunting activities farther in the deep forest. In general, socioeconomic status and wealth are correlated inversely with forest exposure in this region. Subsistence farming, hunting and fishing in the forest are major sources of nutrition for people without formal employment. This observation is in accordance with a study from Nigeria demonstrating an association between low socioeconomic status and loiasis [23].

Loiasis prevalence increases with age based on the fact that it is contracted through progressive accumulation of infections and that the adult worms may live for up to 20 years in the human host [24]. However, the two youngest participants with loiasis were only 5 years of age and were reportedly taken to the rain forest by parents who engaged in twice weekly subsistence agriculture. Females more frequently had loiasis than males (32.2% vs 28.6%, respectively), but this is due to the higher proportion of elderly females participating in the study. Therefore, if adjusted for age the odds ratio of 1 (1-1.1) demonstrates no sex preference for loiasis.

The analysis of risk factors for loiasis in this study population stresses the importance of forest exposure for transmission. Adjusted odds for loiasis increase by a factor of 5.1 (2.6-9.9) with occasional sylvan exposure, 11.1 (5.4-22.6) with frequent sylvan exposure and by 25.7 (12.5-52.9) with intensive sylvan exposure. This highly significant association demonstrates the importance of forest exposure for successful transmission of *L. loa* and points towards potential means for the interruption of its transmission cycle. Inversely, absence of regular activities in the rain forest is a marked protective factor against loiasis (LR - of 0.09). This is consistent with entomological research showing that *Chrysops* flies do not usually come close to human habitats and rarely bite indoors [25]. Similarly, reports from Nigeria and Cameroon showed an association between loiasis and occupational activity in muddy areas and rainforests [26,27] and another study from Gabon showed that agricultural activity and hunting were positvely correlated with loiasis [28]. This is consistent with our findings with regards to the fact that the majority of hunters report intensive forest exposure and those who engage in agricultural activity report any kind of regular weekly forest exposure.

Unspecific symptoms attributable to loiasis are not well understood but pruritus is the most commonly cited complaint [4,8,9]. A study from the Republic of Congo demonstrated that pruritus occurred in 64.4% in a population highly endemic for loiasis [8]. Another Congolese study showed that pruritus was reported as at least “frequent” in 50% (27/50) and 83% (25/30) by two different cohorts with parasitologically confirmed loiasis [9]. A survey from Eastern Cameroon (n = 4146) did not determine the frequency of non-specific pruritus, but showed that among individuals with Calabar swelling pruritus occurred up to 94% [29]. In this study (n = 213) 75% (12/16) of individuals with history of Calabar swelling reported pruritus, but only 28.6% (12/42) of those with pruritus also were positive for history of Calabar swelling. Churchill et al. reported a frequency of pruritus of 27% among African immigrants (14/51) and European expatriates (13/49) who presented at the Hospital of Tropical Diseases in London [4]. Based on this sparse evidence pruritus may be more prevalent in endemic populations than in migrants or travellers and may therefore serve as a clinical predictor for loiasis in Central and West Africa.

The sub-population investigated for Calabar swelling indicated that a proportion of 7.5% (16/213) reported a recent episode of edema suggestive for loiasis. Interestingly, Calabar swelling occurred in only 10 out of 60 participants (16.7%) who met the case definition of loiasis (*P* = 0.002). This rather low proportion of Calabar swelling is consistent with previous reports indicating that this clinical manifestation of loiasis occurs less frequently in persons residing in endemic regions than in travellers [4,7].

### Diagnostic scores

To date, obtaining information on history of eyeworm in its restricted RAPLOA definition is the simplest and most valid method to detect communities of high loiasis burdens [11,12,30]. On individual level highest diagnostic validities, however, may be gained by investigation of multiple, high volume samples of blood, applying concentration techniques and subsequent assessment by microscopy and molecular techniques [2]. This study relied on the assessment of eye worm migration in addition to microscopy of thick smears of 10µl of peripheral blood. It is understood that this diagnostic method may be associated with lower prevalence of microfilaraemia than the above-mentioned methods and therefore constitutes a limitation of the analysis, as potential cases may have wrongly been classified as controls. However, we are confident that these measures are relevant on an epidemiological scale in regions of high *L. loa* transmission. Previous epidemiological studies from Gabon support these findings reporting concordant disease prevalences of 22% and 40% [28,30]. A strength of this survey is the representativeness of the study population due to the absence of any relevant exclusion criteria for study-participation.

Based on forest exposure and pruritus a prediction score was calculated to identify those persons most at risk for loiasis in resource-limited settings, where further laboratory-based testing is challenging. First, absence of regular weekly forest exposure was associated with a negative likelihood ratio of 0.09 corresponding to a large, almost conclusive decrease of disease-likelihood. This translates to a post-test probability of 3.9% for loiasis in a population with a pre-test probability of 30.5%. The combined absence of regular weekly forest exposure and pruritus decreased this already low post-test probability further to 2.4%. Therefore the absence of “regular weekly forest exposure” may help to rule out loiasis in the absence of state-of-the-art diagnostics.

On the opposite the presence of “pruritus” combined with “intensive forest exposure” led to a positive likelihood ratio of 9.18, which constitutes a large and nearly conclusive increase of disease-likelihood. This translates into a post-test probability of 80.1% for loiasis in this study-population and to potentially even higher post-test probabilities in populations of higher loiasis prevalence [31]. It is of mention that areas under the ROC curve for many composite exposures yielded values indicating almost excellent performance.

Based on this score persons most at risk for loiasis may be readily identified by two simple questions about forest exposure and pruritus on a sub-population level in regions of *L. loa* endemicity. This may be particularly useful in the setting of onchocerciasis control programmes as well as in clinical research on loiasis [2]. Finally, individual patients may benefit from such a risk-based approach by employing further laboratory based testing to those with highest probability of loiasis.

## CONCLUSION

Loiasis is a neglected tropical disease that occurs in rural parts of Central and West Africa, where diagnostic tools are often not readily available. This study demonstrates that increasing degrees of sylvan exposure are marked risk factors for loiasis and provides diagnostic composite scores for the estimation of disease-risk. In regions of high *L. loa* endimicity these scores may be used to rapidly identify groups with highest likelihood for loiasis. High-risk individuals may constitute an epidemiological key group in the infection cycle between the disease vector and the human host and may therefore play a crucial role in the transmission of *L. loa*. It is this group of individuals which should be targeted for further diagnostic evaluation to start controlling loiasis in endemic regions of Central and West Africa. Further, the present diagnostic scores may aid policy-makers to tailor the delivery of ivermectin mass drug administration for onchocerciasis control programmes more effectively and safely in regions of high *L. loa* burden.
